# Comparison of stapled haemorrhoidopexy with traditional excisional surgery for haemorrhoidal disease (eTHoS): a pragmatic, multicentre, randomised controlled trial

**DOI:** 10.1016/S0140-6736(16)31803-7

**Published:** 2016-11-12

**Authors:** Angus J M Watson, Jemma Hudson, Jessica Wood, Mary Kilonzo, Steven R Brown, Alison McDonald, John Norrie, Hanne Bruhn, Jonathan A Cook, eTHoS study group

**Affiliations:** a Raigmore Hospital, Inverness, Scotland, UK; b Centre for Healthcare Randomised Trials, University of Aberdeen, Aberdeen, UK; c Health Economics Research Unit, University of Aberdeen, Aberdeen, UK; d Sheffield Teaching Hospitals, Sheffield, UK; e Centre for Statistics in Medicine, University of Oxford, Oxford, UK

## Abstract

**Background:**

Two commonly performed surgical interventions are available for severe (grade II–IV) haemorrhoids; traditional excisional surgery and stapled haemorrhoidopexy. Uncertainty exists as to which is most effective. The eTHoS trial was designed to establish the clinical effectiveness and cost-effectiveness of stapled haemorrhoidopexy compared with traditional excisional surgery.

**Methods:**

The eTHoS trial was a large, open-label, multicentre, parallel-group, pragmatic randomised controlled trial done in adult participants (aged 18 years or older) referred to hospital for surgical treatment for grade II–IV haemorrhoids. Participants were randomly assigned (1:1) to receive either traditional excisional surgery or stapled haemorrhoidopexy. Randomisation was minimised according to baseline EuroQol 5 dimensions 3 level score (EQ-5D-3L), haemorrhoid grade, sex, and centre with an automated system to stapled haemorrhoidopexy or traditional excisional surgery. The primary outcome was area under the quality of life curve (AUC) measured with the EQ-5D-3L descriptive system over 24 months, assessed according to the randomised groups. The primary outcome measure was analysed using linear regression with adjustment for the minimisation variables. This trial is registered with the ISRCTN registry, number ISRCTN80061723.

**Findings:**

Between Jan 13, 2011, and Aug 1, 2014, 777 patients were randomised (389 to receive stapled haemorrhoidopexy and 388 to receive traditional excisional surgery). Stapled haemorrhoidopexy was less painful than traditional excisional surgery in the short term and surgical complication rates were similar between groups. The EQ-5D-3L AUC score was higher in the traditional excisional surgery group than the stapled haemorrhoidopexy group over 24 months; mean difference −0·073 (95% CI −0·140 to −0·006; p=0·0342). EQ-5D-3L was higher for stapled haemorrhoidopexy in the first 6 weeks after surgery, the traditional excisional surgery group had significantly better quality of life scores than the stapled haemorrhoidopexy group. 24 (7%) of 338 participants who received stapled haemorrhoidopexy and 33 (9%) of 352 participants who received traditional excisional surgery had serious adverse events.

**Interpretation:**

As part of a tailored management plan for haemorrhoids, traditional excisional surgery should be considered over stapled haemorrhoidopexy as the surgical treatment of choice.

**Funding:**

National Institute for Health Research Health Technology Assessment programme.

## Introduction

Haemorrhoids are swellings of the sub mucosal veins at the top of the anal canal. Symptoms from haemorrhoids include bleeding, pain, prolapse and peri-anal itch with prevalence rates of up to 44% within the general population.^1^, ^2^ A substantial proportion of the population will have symptoms of haemorrhoids within their lifetime and the presence of per-rectal bleeding, and its association with colorectal cancer, can cause anxiety.^2^

The initial management of haemorrhoids is community based, where no concern exists about the presence of colorectal cancer or inflammatory bowel disease. Dietary manipulation, bulk forming laxatives, and advice should be offered first. Persistent symptoms merit referral for investigation and treatment. Outpatient treatment principally involves rubber band ligation for lower grade haemorrhoids, whereas surgical interventions are used for higher grade haemorrhoids where banding has been unsuccessful. Rubber band ligation is a clinic-based procedure where a small band is placed at the top of a haemorrhoid to reduce its size by interfering with tissue blood supply. The widely adopted Goligher system^3^ for grading haemorrhoids was used in this trial.

Given the prevalence of the condition, the management of haemorrhoidal disease continues to have considerable workload and costs implications for the National Health Service (NHS), with approximately 25 000 haemorrhoidal procedures being performed as hospital day-case or inpatient admissions in England in 2006–07.^4^ Over the past two decades, understanding of the anatomy of haemorrhoids has improved, leading to the introduction of new surgical technologies into clinical practice, often without previous robust assessment. These technologies included stapled haemorrhoidopexy and haemorrhoidal artery ligation, variants of which are promoted through surgical technology industries. The purported advantages of the new treatments, when compared with an existing surgical technique, traditional excisional surgery (or haemorrhoidectomy), were less postoperative pain with similar symptom control.^5^

**Research in context**

**Evidence before this study**

A Health Technology Assessment evidence synthesis and six systematic reviews published between 2006 and 2015 have assessed the role of stapled haemorrhoidopexy versus traditional excisional surgery. Over 50 randomised controlled trials have been conducted of variable size and quality. These studies have suggested short-term pain was higher with traditional excisional surgery than stapled haemorrhoidopexy though recurrence was also higher with stapled haemorrhoidopexy. Previous economic assessments of these operations were based on limited quality of life data, and suggested a shorter operation time for stapled haemorrhoidopexy than traditional excisional surgery. There was also a paucity of medium-to-long-term clinical and economic data regarding stapled haemorrhoidopexy or traditional excisional surgery particularly for grade II haemorrhoids.

**Added value of this study**

We did a large multicentre, open-label, randomised controlled trial of 777 patients comparing stapled haemorrhoidopexy with traditional excisional surgery. To our knowledge, this is the largest trial of this treatment comparison for haemorrhoids. The overall quality of life was significantly better for the traditional excisional group than the stapled haemorrhoidopexy group over 24 months (−0·073 [95% CI −0·140 to −0·006]; p=0·0342). Participants in the traditional excisional surgery group had fewer symptoms at 12 and 24 months (both p<0·0001) and reported fewer recurrences at 12 (39/278) and 24 months (76/300) compared with the stapled haemorrhoidopexy group (94/295 at 12 months and 134/317 at 24 months). Rates of continence and tenesmus were better in the traditional excisional surgery group than in the stapled haemorrhoidopexy group; complications were similar in both groups. Pain and analgesic use were lower in the stapled haemorrhoidopexy group in the first 3 weeks after surgery, but time to return to normal activity 6 weeks after surgery was similar between groups. No difference was observed in length of stay or operating time between groups. Traditional excisional surgery was found to be more cost-effective than stapled haemorrhoidopexy terms of cost-effectiveness.

**Implications of all the available evidence**

The results of this study show that although both stapled haemorrhoidopexy and traditional excisional surgery are equally safe, traditional excisional surgery has better quality of life and is associated with fewer symptoms and recurrence of haemorrhoids. Additionally, stapled haemorrhoidopexy is more expensive and is not cost-effective.

Despite numerous small-scale randomised controlled trials, significant doubts remain about the usefulness, efficiency, and cost-effectiveness of stapled haemorrhoidopexy and haemorrhoidal artery ligation.^6^ Evidence synthesised in systematic reviews and Health Technology Assessments^7^, ^8^, ^9^, ^10^, ^11^, ^12^, ^13^ has highlighted the lack of good quality data on which to base management choices. This led to the National Institute of Health Research commissioning two trials to perform robust assessments of the newer techniques (eTHoS and haemorrhoidal artery ligation *vs* rubber band ligature for the management of symptomatic grade II and III haemorrhoids [HubBLe]). The HubBLe trial recently reported its results which compared haemorrhoidal artery ligation with rubber band ligature. Although a small overlap exists between HubBLe and eTHoS, the trials were designed to dovetail together with less advanced disease falling mainly into the HubBLe trial and more advanced disease eligible for eTHoS. The authors of HubBLe concluded that if rubber band ligature was considered as a course of treatment, recurrence rates of haemorrhoids were similar to that with haemorrhoidal artery ligation, and haemorrhoidal artery ligation was more expensive and not cost-effective.^14^ The aim of the eTHoS trial was to assess whether stapled haemorrhoidopexy was more effective and cost-effective compared with traditional excisional surgery in the treatment of grade II, III, and IV haemorrhoids. The primary objective was to compare health-related quality of life derived over 24 months.

## Methods

### Study design and participants

The eTHoS trial was a large, open-label, multicentre, parallel-group, pragmatic randomised controlled trial done in 32 UK NHS hospitals. Potential participants were adults aged 18 years or older referred to hospital for surgical treatment for haemorrhoids. Patients with haemorrhoids (grade II–IV), who provided written informed consent, were eligible to take part. Participants referred to hospital with haemorrhoids were included in the trial if their symptoms were refractory to rubber band ligature or haemorrhoidal artery ligation or if their haemorrhoids were thought to be too large for these treatments to be successful. Those precluded from the trial included patients who had previous surgery for haemorrhoids (traditional or stapled) and those who had previous surgical treatment for anal sphincter injury repair, or had symptomatic incontinence or peri-anal sepsis. Those with known inflammatory bowel disease or malignant gastrointestinal disease, within the past 5 years, or who were deemed medically unfit for surgery, were also ineligible, as were pregnant women. The exclusion criteria were set to have a minimum effect on the overall numbers of participants recruited to the trial and hence maximise generalisability. The study was approved by the North of Scotland Research Ethics Committee on June 18, 2010 (reference number 10/20802/17). The protocol was published in 2014.^15^

### Randomisation and masking

Participants were randomly assigned (1:1) to receive either traditional excisional surgery or stapled haemorrhoidopexy with the use of an automated system with telephone and web-based interfaces run from the trial office. The randomisation minimisation algorithm used included centre, grade of haemorrhoidal disease (II, III, or IV), baseline EuroQol 5 dimensions 3 level (EQ-5D-3L) descriptive system score,^16^ and sex. Patients and investigators were not masked to treatment allocation.

### Procedures

Participants were recruited by surgeons and research nurses in the outpatient department. The surgeons and research nurses were also responsible for initiating randomisation. Eligible and consented participants were placed on the appropriate waiting list by the treating colorectal surgeon or a designated team member. The EQ-5D-3L UK wording and population norms (range −0·59 to 1·0, with 1·0 being optimum),^16^ 36-item Short Form Health Survey (SF-36) version 2 score, Cleveland incontinence score (CIS; range 0–20, with 0 being optimum), and Haemorrhoid symptom score (HSS; 0–26 range, with 0 being optimum) were collected pre-randomisation as baseline measures.^17^, ^18^ Other data collected at baseline included height, weight, grade of haemorrhoid, anticoagulant medication prescriptions, and previous treatments for haemorrhoids. Each centre's participating surgeons had received appropriate recognised training for both stapled and traditional haemorrhoid surgery and were using the procedures routinely in their hospitals. Surgery was only done by surgeons in the late stage of training if they had been signed off by their supervising consultant surgeon, or if they were operating under the direct supervision of their consultant. Preoperative and postoperative care followed the respective surgeon's and NHS hospital's standard policies. Stapled haemorrhoidopexy aims to correct haemorrhoidal prolapse by excising a ring of tissue above the haemorrhoidal cushions with immediate re-anastomosis of the mucosa with the use of staples. A secondary effect might be to reduce blood flow and therefore congestion. Fibrosis develops at the staple line maintaining the haemorrhoids in their new position. Stapled haemorrhoidopexy is done with the use of a stapling gun. Three haemorrhoid stapling devices are commonly in use within the UK (Johnson & Johnson, Chex, and Covidien). Reflecting the pragmatic nature of the trial, surgeons were able to use the gun which they normally use in their routine practice.

There are two commonly used traditional excisional procedures done across the world: open^19^ and closed.^20^ Both have the intention of excising the haemorrhoidal cushions. The procedure is most commonly done with electrocautery. In this trial, surgeons undertook whichever procedure they would do as part of their routine practice. The use of the Ligasure Medtronic (Minneapolis, MN, USA) and Harmonic Ethicon Johnson and Johnson (NJ, USA) devices to perform an excisional haemorrhoidectomy were excluded in this trial. On the day of surgery, the grade of surgeon and anaesthetist and the type of anaesthesia and the surgical technique were recorded. Additionally, the length of time the procedure took and any intraoperative complications were recorded. Data for postoperative complications before discharge, which included postoperative bleeding, pelvic sepsis, the need for blood transfusion, and urinary retention were collected.

Participants were followed up for 24 months; this consisted of a single clinic visit followed by multiple postal questionnaires completed by the participant. Routine data were also used to measure recurrence and further treatment. At the 6 week clinic appointment, data for postoperative complications including haemorrhage, requirement for blood transfusion, anal stenosis, anal fissure, urinary retention (which required catheterisation), residual anal skin tags, difficult defecation, wound discharge, pelvic sepsis, and pruritus were collected. Clinical examination of the anal canal was not routinely performed. Further haemorrhoid-related interventions since discharge and the need for further planned medical and/or surgical treatments for haemorrhoids or complications associated with treatment were recorded. Postoperative examination of the anal canal during the 6 week follow-up clinic was included in the protocol but was not done routinely if patients reported that their symptoms were improving. This is in line with current practice and was also adhered to in the HubBLe trial.

EQ-5D-3L and visual analogue scale (VAS) pain data were collected by postal questionnaire at 1 and 3 weeks after surgery. EQ-5D-3L, SF-36, HSS, CIS, and VAS data were collected by postal questionnaire at 6 weeks after surgery. Additionally, questionnaires distributed at 12 and 24 months after randomisation also collected data for patient-reported haemorrhoid recurrence and further operations. Participants who did not respond to 12 and 24 month questionnaires were sent a postal reminder and a shortened version of the questionnaire containing the EQ-5D-3L only. The main outcome assessment was planned at 24 months (from the date of randomisation) follow-up. Consent was sought from all participants to be flagged for notification of haemorrhoidal recurrence. To assess long-term safety, the participants were investigated for further haemorrhoidal surgery through Hospital Episode Statistics (HES) in England, Patient Database Wales (PEDW) in Wales, and Information Services Division (ISD) data in Scotland, when all participants had reached 12 months post-randomisation.

### Outcomes

The primary outcome was the area under the quality of life curve (AUC) over 24 months derived from EQ-5D-3L measurements taken from patient questionnaires distributed at baseline, 1 week, 3 weeks, 6 weeks (postoperative), 12 months, and 24 months post-randomisation. The AUC is expressed in years and can be interpreted as quality-adjusted life-years (QALYs). The primary trial economic outcome was incremental costs per QALY gained with QALYs based on the responses to the EQ-5D-3L over 24 months. Patient-reported secondary outcomes were generic health profile measured by SF-36 and EQ-5D-3L, VAS pain score, CIS, HSS, postoperative analgesia consumption, recurrence of haemorrhoids, and tenesmus. Tenesmus can be a disabling symptom which is described as a feeling of wanting to pass stool, even though the rectum is empty. The clinical secondary outcomes were further interventions, intraoperative and postoperative complications including haemorrhage, requirement for blood transfusion, anal stenosis, anal fissure, urinary retention (which required catheterisation), residual anal skin tags, difficult defecation, wound discharge, pelvic sepsis, and pruritus. A serious adverse event was defined as an event occurring to a research participant that was treatment related (resulted from administration of any of the research procedures) and was expected or unexpected that caused death, was life threatening, required hospital admission, resulted in significant incapacity or disability, or was otherwise considered medically significant by the investigators. Death, related or not to the study treatment, was also recorded.

### Statistical analysis

A sample size of 338 per group was required to provide 90% power to detect a difference in the mean area under the quality of life AUC curve of 0·25 SDs derived from EQ-5D-3L score measurements, with a significance level of 5% (two-sided α). Data for the EQ-5D-3L AUC, in this patient group, were limited at the time of study conduct but an SD of 0·25 was thought to be sufficient to detect a worthwhile difference in quality of life measures. To allow for 15% non-response in the outcome, we randomly assigned 400 patients in each of the two groups. Such a sample size would also provide 90% power to assess differences in the secondary outcome of recurrence between the two surgical techniques from around 10% to around 4%. This magnitude of difference was supported by a systematic review,^9^ which showed a higher recurrence in the stapled haemorrhoidopexy group compared with the traditional excisional surgery group, but this was not statistically significant. No adjustment in sample size was made for the potential gain in precision due to adjustment for the baseline EQ-5D-3L score, haemorrhoid grade, or sex.

Study analyses followed a comprehensive, prespecified statistical analysis plan. The main statistical analyses were based on all participants as randomised, irrespective of subsequent compliance with treatment allocation. Prespecified subgroup analyses investigated the influence of haemorrhoidal grade and sex with the use of treatment by subgroup interaction effects. The primary outcome measure was analysed with linear regression adjusting for the other minimisation variables as fixed effects with the exception of centre, which was accounted for using cluster robust standard errors. The preplanned principal analysis included participants with at least one short-term post-surgery follow-up (1, 3, or 6 weeks) and at least one long-term measurement (12, 18, or 24 months), to calculate the AUC. Sensitivity analyses included all participants with a long-term measure. Secondary analyses of the primary outcomes explored sensitivity to assumptions regarding interpolation, missing data (using multiple imputation by chained equations using the “ice” Stata command^21^ to impute the missing EQ-5D-3L scores 30 times before calculating the AUC and then using Rubin's rule to combine and estimate), and also compliance (using a per-protocol population consisting of those who received the allocated intervention). The EQ-5D-3L AUC over 6 weeks and 12 months was also compared. The secondary outcome tenesmus was analysed with the χ^2^ test for trend, further surgical intervention was analysed with the χ^2^ test. The other secondary outcomes were analysed with generalised linear models (ie, logistic regression) or linear mixed models with adjustment for the minimisation variables. Continuous variables were summarised with mean (SD) or median (IQR) whereas discrete variables were reported as absolute number and percentage in each category. All analyses were assessed at the two-sided 5% significance level except for the subgroups which were assessed at two-sided 1% level.

A cost-effectiveness analysis in terms of incremental cost per QALYs gained was undertaken. The costs of the interventions were estimated by identifying, measuring, and valuing resource use. Resource use was identified and measured from case report forms and patient-reported questionnaires and was valued with published source such as NHS reference costs.^22^ All costs were estimated from the NHS and personal social perspective as per National Institute for Health and Care Excellence recommendations. As patients were followed up for more than a year, costs and QALYs were discounted at the recommended rate of 3·5%.^23^ The economic data analysis accounted for missing data using multiple imputation. Both the statistical and the economic analyses were done using Stata, version 14. This trial is registered with the ISRCTN registry, number ISRCTN80061723.

### Role of the funding source

The funder of the study had no role in study design, data collection, data analysis, data interpretation, or writing of the report. The corresponding author had full access to all the data in the study and had final responsibility for the decision to submit for publication.

## Results

**Figure 1:**
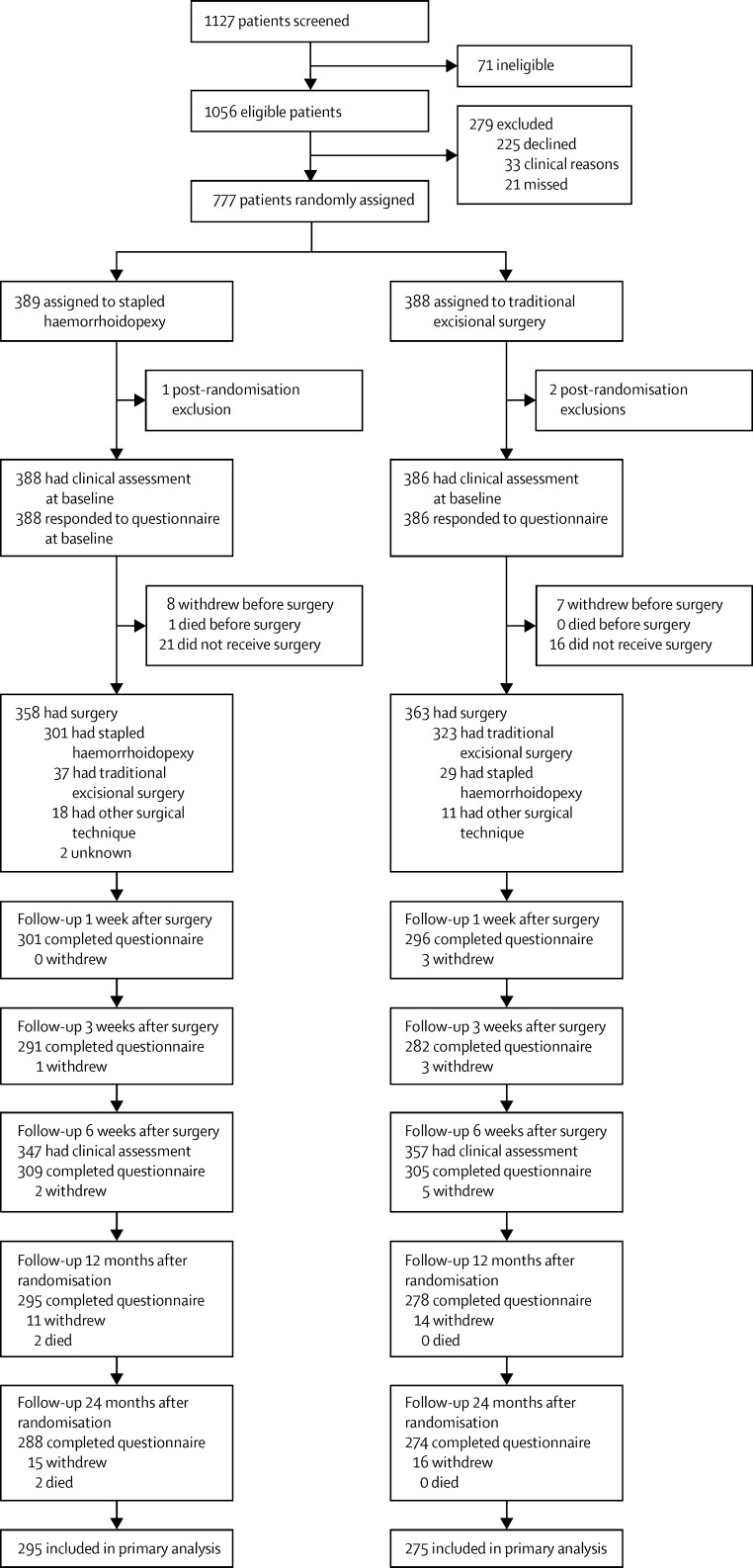
Trial profile Numbers of participants declining further follow-up or not responding to the questionnaire are cumulative in direction of participant flow.

Between Jan 13, 2011, and Aug 1, 2014, 1127 patients were screened for eligibility. Of these, 777 were randomly assigned to either receive stapled haemorrhoidopexy (n=389) or traditional excisional surgery (n=388; figure 1). 774 participants were included in the analysis as three participants were excluded after randomisation; one from the stapled haemorrhoidopexy group and two from the traditional excisional surgery group. The trial was terminated on July 8, 2016. Median follow-up was 731 days (IQR 377–736) for the stapled haemorrhoidopexy group and 731 days (514–738) for the traditional excisional surgery group. Baseline questionnaire and clinical case report form data were available for all randomised participants (table 1). 721 participants received surgery, and, of these, 37 participants in the stapled haemorrhoidopexy group received traditional excisional surgery and 29 in the traditional excisional surgery group received stapled haemorrhoidopexy. The two main reasons for not receiving randomised treatment were surgeons' decision after clinical examination and participant preference for surgery.

272 (84%) of 323 participants in the traditional excisional surgery group underwent the Milligan Morgan technique for excisional surgery. Most procedures were done under general anaesthesia (341 [95%] of 358 in the stapled haemorrhoidopexy group *vs* 350 [96%] of 363 in the traditional excisional surgery group), some with an additional local anaesthetic block (105 [29%] in the stapled haemorrhoidopexy group *vs* 127 [35%] in the traditional excisional surgery group). 256 (72%) of 358 participants were operated on by consultant surgeons in the stapled haemorrhoidopexy group compared with 225 (62%) of 363 in the traditional excisional surgery group. Surgical trainees performed 58 (16%) of 358 stapled haemorrhoidopexy procedures and 89 (25%) of 363 traditional excisional surgeries. Specialty doctors performed the remaining operations (32 [9%] of 358 in the stapled haemorrhoidopexy group *vs* 37 [10%] of 363 in the traditional excisional surgery group). No differences were noted between the duration of the operation and the length of stay; time spent waiting for surgery was also similar between groups (table 1).

**Figure 2:**
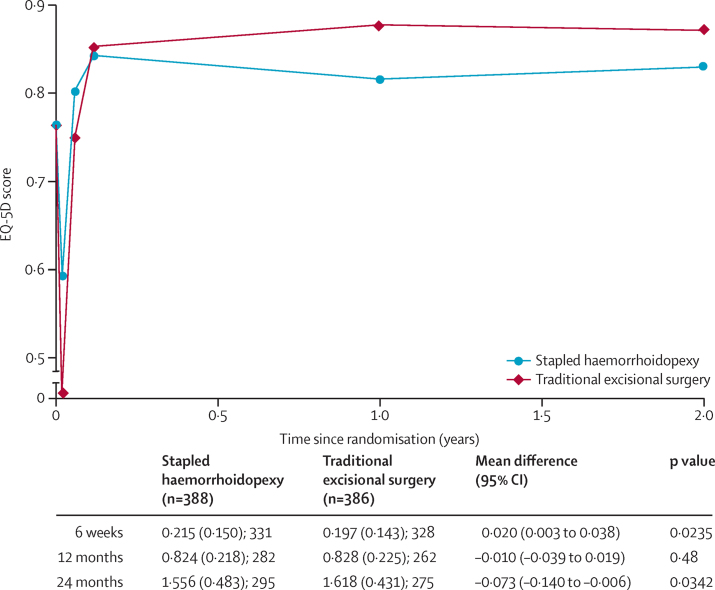
AUC EQ-5D-3L score comparison between stapled haemorrhoidopexy and traditional excisional surgery groups Participants need to have at least one short-term and one long-term follow-up score for inclusion in the primary analysis. Mean (SD); N is shown for 6 weeks, 12 months, and 24 months. ED-5D-3L AUC=EuroQoL 5 dimensions 3 level area under a curve. A breakdown of the number of participants included at each timepoint in the analysis is given in the appendix (p 3).

The EQ-5D-3L profile for the two treatment groups is shown in figure 2. Participants in the stapled haemorrhoidopexy group had higher EQ-5D-3L scores than those in the traditional excisional surgery group at 1 and 3 weeks whereas the EQ-5D-3L scores were higher in the traditional excisional surgery group than the stapled haemorrhoidopexy group from 6 weeks onwards. The primary outcome, EQ-5D-3L AUC over 24 months was higher in the traditional excisional surgery group: mean difference −0·073 (95% CI −0·140 to −0·006); the AUC over 12 months post-randomisation showed no difference between the two interventions. Secondary analyses of EQ-5D-3L AUC at 24 months, which explored sensitivities to assumptions (per protocol) and multiple imputation analyses (appendix p 1), were consistent with the main analyses for all time horizons. Subgroup analyses of sex and grade of haemorrhoid did not reveal treatment effect differences in EQ-5D-3L AUC (appendix p 1). Both the physical and mental components of the SF-36 improved after surgical intervention, with participants in the traditional excisional surgery having significantly more improvement when compared with stapled haemorrhoidopexy at 12 months (table 2). This difference was sustained at 24 months, but was not statistically significant.

While participants in the stapled haemorrhoidopexy group had a similar HSS score to the traditional excisional surgery group at 6 weeks (mean difference 0·15, 95% CI −0·60 to 0·91; p=0·69), it was higher at both 12 months (2·09, 1·28–2·90; p<0·0001), and 24 months (1·46, 0·64–2·28; p=0·0005; table 2). Additionally, 295 participants in the stapled haemorrhoidopexy group and 278 in the traditional excisional surgery group responded to a question about whether their haemorrhoids had come back 12 months after randomisation. In the stapled haemorrhoidopexy group, 94 (32%) of 295 participants reported that their symptoms had returned compared with 39 (14%) of 278 in the traditional excisional surgery group (odds ratio [OR] 2·96; 95% CI 2·02–4·32, p<0·0001), and this difference was maintained at 24 months (table 3). Registry data (ISD/PEDW/HES, of which the following codes were flagged: H511, H513) and self-reports of further surgical interventions were combined. Self-reported and registry data were verified by contacting sites for all but six cases (because of time constraints we were unable to verify four cases and in two additional cases, the sites did not respond to our queries); data was still included if the site did not confirm an intervention was done. If there was discrepancy between self-reported and registry data, self-reported data were used. A higher incidence of further surgery was reported in the stapled haemorrhoidopexy group compared with the traditional excisional surgery group (table 3).

Participant reported pain with VAS was significantly better in the stapled haemorrhoidopexy group at 1 and 3 weeks after surgery than in the traditional excisional surgery group but no difference between groups was noted at 6 weeks (appendix p 2). Analgesia use at 3 weeks was lower in the stapled haemorrhoidopexy group than in the traditional excisional surgery group (OR 0·58, 95% CI 0·45–0·75, p<0·0001), but no difference was reported at 1 and 6 weeks (appendix p 2). At 12 and 24 months after randomisation, a significant difference between CIS between treatment groups was noted; however, the scores were similar in both groups 6 weeks after randomisation (table 2). Participants were asked about the presence of tenesmus at 6 weeks, 12 months, and 24 months. Tenesmus was more prevalent in the stapled haemorrhoidopexy group throughout the follow-up period (table 2).

Serious adverse events were reported by treatment received. 24 (7%) participants had a serious adverse event after undergoing stapled haemorrhoidopexy and 33 (9%) after receiving traditional excisional surgery (table 4). One participant died in the stapled haemorrhoidopexy group, but this death was unrelated to haemorrhoid surgery. Most hospital admissions were for pain, bleeding, constipation, and urinary retention. Only two participants received a blood transfusion (one participant had low concentration of haemoglobin before surgery and one participant was readmitted 1 week after surgery with severe postoperative bleeding, requiring an extensive hospital stay). 11 participants required catheterisation for urinary retention; seven had received traditional excisional surgery and four stapled haemorrhoidopexy. Ten participants remained in hospital or were readmitted with pain in the traditional excisional surgery group compared with six in the stapled haemorrhoidopexy group. A few participants had a combination of pain, constipation, and bleeding, but bleeding on its own was more common in the stapled haemorrhoidopexy group than in the traditional excisional surgery group. Two participants in each group reported pain caused by an anal fissure. No episodes of pelvic sepsis or rectal perforation were recorded in this trial.

The mean cost per patient for stapled haemorrhoidopexy was £941 (SD 415) compared with £602 (507) for traditional excisional surgery (appendix p 3). The adjusted analysis mean difference in total costs was £337 (95% CI 251–423) higher for stapled haemorrhoidopexy than for traditional excisional surgery. The QALY results for the stapled haemorrhoidopexy group were 1·62 (SD 0·43) and 1·69 (0·38) for the traditional excisional surgery group. The adjusted analysis mean difference in QALYs between groups was −0·070 (95% CI −0·127 to −0·011). Overall, stapled haemorrhoidopexy cost more than traditional excisional surgery and had a lower number of QALYs than traditional excisional surgery. The cost utility analysis indicated that stapled haemorrhoidopexy has <0·1% probability of being cost-effective at £20 000 and 0·1% probability of being cost-effective at £30 000 willingness to pay threshold.

## Discussion

The eTHoS trial showed that overall quality of life in the traditional excisional surgery group was better than the stapled haemorrhoidopexy group during the 24 month follow-up. On the basis of previous work on clinically important differences in the EQ-5D-3L score, the magnitude of difference in favour of traditional excisional surgery was clinically important (the equivalent of the quality of life being greater by a clinically important amount, for a full year). The minimum important difference for the EQ-5D-3L has been estimated to be around 0·07 based on an anchor method applied to a range of patient populations.^24^, ^25^ Additionally, participants in the traditional excisional surgery group reported fewer haemorrhoid incontinence symptoms, and recurrences. Stapled haemorrhoidopexy was less painful than traditional excisional surgery, but this difference disappeared by 6 weeks. Short-term quality of life scores after surgery favoured stapled haemorrhoidopexy, reflecting the lower rates of pain in the immediate postoperative period. At 12 months, the EQ-5D-3L quality of life scores were similar between groups, however, the physical and mental health domains of the SF-36 favoured the traditional excisional surgery group. At 24 months, the primary outcome favoured traditional excisional surgery. Additionally, less residual haemorrhoid symptoms and fewer recurrences and re-interventions were noted in the traditional excisional surgery group.

No differences in the operating time, length of stay, or return to normal activity at 6 weeks were noted between the two procedures. This refutes the common argument that a shorter operating time and length of stay after stapled haemorrhoidopexy offsets the cost of a stapler and, taken alone, draws in to question the continued use of the technique. The continued use of stapled haemorrhoidopexy is further drawn into question by the other findings indicating a higher recurrence rate, more tenesmus, a higher cost, worse continence and equivocal complications. Even the reduced pain after stapled haemorrhoidopexy was equivalent to that in the traditional excisional surgery group 6 weeks postoperatively.

Clinical recurrence of haemorrhoids was measured using HSS, a patient-reported dichotomous outcome measure, and recurrence data from national databases (ISD, HES, PEDW). Although the HSS has not been formally validated, the rationale for its use has been previously articulated^14^ and at the inception of the trial it was the best assessment tool available. We chose to assess recurrence over 24 months expecting to capture a proportion of patients who report symptom relapse after 12 months, and we found this to be the case. Little data are available concerning the long-term success rates of haemorrhoid surgery and after 24 months, distinguishing between symptoms caused by inadequate initial treatment and the occurrence of new disease would be important.^10^

Continence was an important outcome measure for this trial. The anal sphincter complex is comprised of two concentric muscles. Anal cushions (which form the basis of haemorrhoids) in the upper part of the canal contribute to continence by acting as washers helping to form a seal. Prolapsing haemorrhoids, therefore, interfere with this mechanism by disrupting the sealing mechanism of the sphincters and cushions working in concert. The traditional excisional surgery technique involves removing tissue close to the internal sphincter. Damage and therefore impairment of continence is widely reported after traditional excisional surgery.^26^ The optimum technique for stapled haemorrhoidopexy involves the accurate placement of the staple line 3–4 cm from the anal verge and therefore above the dentate line in the columnar mucosa of the anal canal. Staple lines lower than this might encroach on the more sensate squamous mucosa of the anal canal contributing to an increase in postoperative pain and a greater chance of injuring the internal sphincter.^27^ As expected, in the immediate postoperative period, CIS was impaired in both groups at 6 weeks when compared with baseline scores, explained by the presence of pain and healing. Thereafter, continence scores were significantly improved in the traditional excisional surgery group up to 24 months after randomisation. High quality surgery and the avoidance of sphincter injury in both groups, and a reduction in the volume of haemorrhoid tissue, could explain the slight improvement in continence scores over the course of the trial.

The economic analysis showed that traditional excisional surgery cost £337 less and had 0·07 more QALYs than the stapled haemorrhoidopexy group over the 24 month follow-up period. Stapled haemorrhoidopexy has a 0·1% probability of being considered cost-effective at the £30 000 threshold. Taken together with the HubBLe health-care cost analysis, both trials report that neither of the newer surgical techniques are cost-effective. The results of the economic analysis differ from those published in the study by Burch and colleagues.^7^ In their study, Burch and colleagues reported that traditional excisional surgery and stapled haemorrhoidopexy had similar costs and QALYs, but that the costs of the staple gun were offset by savings in hospital stay. In our study, the inpatient stay was similar across both groups, so no cost saving in inpatient stay was reported, and our QALY results, on the basis of a 24 month follow-up, indicated that stapled haemorrhoidopexy had lower QALYs than traditional excisional surgery. A paucity of robust economic data is available on haemorrhoid surgery, however, if the results of these contemporaneous trials are put into practice, substantial annual savings in publicly funded health services could be achieved.

Several decisions were made reflecting the pragmatic nature of the study's conception and design and the aim to reflect routine care. First, the study was open label in that no attempt was made to mask participants (who were generally the outcome assessor) or the surgeon to treatment allocation. The presence or absence of peri-anal wounds would render masking impossible in the short term. No prescriptive entry criteria were set for hospital inclusion and we are therefore confident that the results are generalisable across health services. A 24 month follow-up was used to capture symptom recurrence and further interventions; therefore, a median follow-up of 731 days was a further strength of this study. A pragmatic approach to surgeons' credentials was used. At the inception of the trial, both techniques were established in common surgical practice and surgeons must have undergone appropriate recognised training for both procedures. Ideally, this will have included attendance at a masterclass. The effect of surgical experience on outcomes can be partly mitigated by the high level of consultant involvement in performing the surgery and the low incidence of adverse events.

The trial had several limitations. As noted previously, participants and outcome assessors were not masked to treatment assignment which could have led to bias when measuring the subjective outcomes. Final recruitment to eTHoS was slightly short of the total target of 800 participants and, furthermore, there were substantial, and greater than anticipated, missing data at 24 months follow-up despite multiple strategies to mitigate this. This, perhaps, reflected the population (working age), the condition (chronic and considered by some to be a sensitive subject), and the nature of the follow-up. Nevertheless, the study still had sufficient statistical precision to detect differences between treatment groups. Various secondary analyses explored plausible imputation and missing data assumptions regarding quality of life and a consistent pattern of benefit in favour of traditional excisional surgery was reported. A noticeable amount of non-compliance with allocation (some not receiving surgery, and some receiving a different operation) was noted reflecting perhaps a mixture of clinical reality and also some surgeon and patient preferences regarding treatment. Such non-compliance tends to dilute a genuine effect. We assessed the effect of non-compliance in the per-protocol population (appendix p 1). Despite this, the primary analysis still supported a difference in favour of traditional excisional surgery, and the per-protocol analysis of only those who complied with allocation was consistent with this finding. The delivery of the interventions reflected routine clinical practice across a range of centres in terms of the surgeons participating (their experience) and how the interventions were delivered (specific technique, centre practices). Comparison of outcome between surgeons and surgical practice was difficult in this context because of the numerous factors which affect patients and centres which were not attributable to the surgeon. Taken together, we believe the findings are robust and generalisable.

The interventions compared in eTHoS reflected clinical practice in the UK NHS at the time of its design. During the recruitment period a new intervention, haemorrhoidal artery ligation, started being used. The HubBLe trial showed no benefit for haemorrhoidal artery ligation over rubber band ligation.^14^ Together with eTHoS, these studies suggest a pattern of failure of new and purported better interventions to achieve sufficient clinical outcome at an appropriate cost. They provide a warning about the widespread adoption of expensive and unproven new procedures. The IDEAL framework^28^, ^29^, ^30^ provides a potential pathway from idea to robust assessment, though to date, while perhaps improving, surgical assesement is still often too late and not rigorous enough.

The introduction of new surgical treatments for haemorrhoids has been accompanied by the greater incidence of reporting of adverse events. Some of the published postoperative complications after surgery for a benign condition, have been severe, particularly with regard to pelvic sepsis, rectal perforation, and rectovaginal fistula formation.^31^ There were no reports of these complications within this trial, which might reflect safe surgical practice within trial centres or that complications occur more frequently within the earlier part of surgical learning curves. Serious adverse events were equally distributed in both groups. All the events were expected and largely consisted of pain, bleeding, constipation, and urinary retention.

The findings in this trial can be compared with a recent network meta-analysis,^10^ which included 98 trials of procedures for grade III and IV haemorrhoids in the analysis. The authors suggested that traditional excisional surgery was associated with fewer haemorrhoid recurrences and that stapled haemorrhoidopexy was associated with less postoperative pain and a higher rate of recurrence. However, the eTHoS trial refutes the data on higher complications rates, return to normal activity, length of operation, and length of stay in traditional excisional surgery. Taken together, the HubBLe and eTHoS trials, also answer the authors call for further high quality randomised controlled trials incorporating economic cost comparisons, and an updated network meta-analysis incorporating these two large trials would be appropriate.

Overall, traditional excisional surgery is both more clinically effective and less costly when compared with stapled haemorrhoidopexy. It is more painful in the short term but this pain can be adequately managed at home. Time to return to normal activity was similar in both groups. In addition to superior quality of life measures, HSS, continence, and tenesmus rates and the need for further surgery were all lower with traditional excisional surgery. Traditional excisional surgery is, therefore, a superior surgical treatment to stapled haemorrhoidopexy for the management of grade II–IV haemorrhoids.

**This online publication has been corrected. The corrected version first appeared at thelancet.com on October 26, 2016**

## Figures and Tables

**Table 1:** Baseline characteristics and operative details

		**Stapled haemorrhoidopexy (n=388)**	**Traditional excisional surgery (n=386)**
**Baseline characteristics**			
Age (years)		50 (40–60)	49 (40–59)
Sex			
	Male	201 (52%)	197 (51%)
	Female	187 (48%)	189 (49%)
Body-mass index		27·0 (5·2); 372	27·0 (4·9); 367
Grade of haemorrhoid			
	II	86 (22%)	86 (22%)
	III	243 (63%)	240 (62%)
	IV	59 (15%)	60 (16%)
Previous haemorrhoid treatment*		139 (36%)	116 (30%)
Pain (visual analogue score)†		2·8 (2·7); 379	2·5 (2·6); 383
EQ-5D-3L‡		0·764 (0·264); 388	0·762 (0·246); 386
Cleveland incontinence score		4·3 (3·9); 376	4·1 (4·0); 376
Haemorrhoid symptom score		10·8 (4·7); 370	10·4 (4·7); 370
**Operative details**			
Duration of operation (h)		0·4 (0·2); 346	0·4 (0·2); 359
Length of hospital stay (days)		0·4 (0·3); 356	0·4 (0·4); 363
Day cases		305/356 (86%)	317/363 (87%)
Time to surgery (days)		68·2 (78·3); 358	68·8 (81·1); 363

Data are median (IQR), n (%), or mean (SD); N. EQ-5D-3L=EuroQol 5 dimensions 3 level.

* Previous haemorrhoid treatment refers to rubber band ligature or haemorrhoidal artery ligation.

† Scale range is from 0 (no pain) to 10 (worst imaginable pain).

‡ The EQ-5D-3L index ranges from −0·59 to 1·0, with positive values indicating a perfect outcome and negative values imply states worse than death.

**Table 2:** Secondary outcomes

			**Stapled haemorrhoidopexy (n=388)**	**Traditional excisional surgery (n=386)**	**Effect size**	**95% CI**	**p value**
**Cleveland incontinence score**							
Baseline			4·3 (3·9); 376	4·1 (4·0); 376	..	..	..
6 weeks			5·1 (4·5); 288	5·0 (4·3); 290	−0·16	(−0·74 to 0·42)	0·58
12 months			4·4 (4·3); 266	2·9 (3·5); 247	1·20	(0·58 to 1·81)	0·0001
24 months			3·8 (3·8); 249	3·0 (3·4); 235	0·79	(0·15 to 1·42)	0·0149
**Haemorrhoid symptom score**							
Baseline			10·8 (4·7); 370	10·4 (4·7); 370	..	..	..
6 weeks			8·2 (5·1); 288	7·9 (5·0); 291	0·15	(−0·60 to 0·91)	0·69
12 months			6·6 (5·1); 263	4·3 (4·3); 243	2·09	(1·28 to 2·90)	<0·0001
24 months			6·4 (5·0); 250	4·8 (4·4); 240	1·46	(0·64 to 2·28)	0·0005
**SF-36**							
Physical component summary							
	Baseline		48·5 (9·4); 380	48·8 (9·5); 377	..	..	..
	6 weeks		48·2 (10·4); 294	48·9 (9·2); 293	−0·58	(−1·77 to 0·61)	0·34
	12 months		49·7 (10·1); 265	51·2 (9·4); 255	−1·79	(−3·06 to −0·51)	0·0059
	24 months		50·3 (10·1); 250	51·1 (9·4); 234	−1·15	(−2·47 to 0·16)	0·0860
Mental component summary							
	Baseline		48·8 (11·7); 380	49·6 (11·0); 377	..	..	..
	6 weeks		47·3 (12·7); 294	48·7 (11·7); 293	−0·55	(−2·07 to 0·97)	0·48
	12 months		48·8 (12·2); 265	51·2 (10·4); 255	−1·71	(−3·34 to −0·08)	0·0396
	24 months		49·8 (11·2); 250	51·0 (10·9); 234	−0·89	(−2·57 to 0·80)	0·30
**Tenesmus**							
6 weeks							
	Participants followed up		309	305	..	..	0·0010
		Always	17 (6%)	9 (3%)	..	..	..
		Often	42 (14%)	20 (7%)	..	..	..
		Sometimes	82 (27%)	75 (25%)	..	..	..
		Rarely	58 (19%)	68 (22%)	..	..	..
		Never	109 (35%)	131 (43%)	..	..	..
		Missing	1 (<1%)	2 (1%)	..	..	..
12 months							
	Participants followed up		295	278	..	..	<0·0001
		Always	11 (4%)	4 (1%)	..	..	..
		Often	27 (9%)	7 (3%)	..	..	..
		Sometimes	60 (20%)	46 (17%)	..	..	..
		Rarely	65 (22%)	43 (15%)	..	..	..
		Never	113 (38%)	154 (55%)	..	..	..
		Missing	19 (6%)	24 (9%)	..	..	..
24 months							
	Participants followed up		288	274	..	..	0·0012
		Always	6 (2%)	3 (1%)	..	..	..
		Often	27 (9%)	13 (5%)	..	..	..
		Sometimes	51 (18%)	36 (13%)	..	..	..
		Rarely	55 (19%)	47 (17%)	..	..	..
		Never	119 (41%)	143 (52%)	..	..	..
		Missing	30 (10%)	32 (12%)	..	..	..

Data are n (%) or mean (SD); n. 6 weeks refers to time since sugery and 12 months and 24 months refers to time since randomisation. Effect sizes are mean differences. SF-36=36-item Short Form Health Survey. Always=one or more times daily. Often=more than once a week but less than once daily. Sometimes=more than once a month but less than once a week. Rarely=less than once a month. Missing=question not completed.

**Table 3:** Further surgical interventions and patient-reported recurrence

			**Stapled haemorrhoidopexy (n=388)**	**Traditional excisional surgery (n=386)**	**Odds ratio (95% CI)**	**p value**
Further surgical intervention 24 months after randomisation			34/364 (9%)	23/371 (6%)	..	0·11
	Patients with registry data*		14	12	..	..
		Patients with self-reported data*	26	15	..	..
Patient-reported recurrence						
	12 months after randomisation					
		Haemorrhoids reoccurred	94/295 (32%)	39/278 (14%)	2·96 (2·02–4·32)	<0·0001
	24 months after randomisation					
		Haemorrhoids reoccurred	134/317 (42%)	76/300 (25%)	2·25 (1·46–3·46)	<0·0001

Data are n/N (%), unless otherwise specified.

* Some participants had both registry and self-reported data.

**Table 4:** Serious adverse events by treatment received

		**Stapled haemorrhoid-opexy (n=338)**	**Traditional excisional surgery (n=352)**
Participants who had a serious adverse event		24 (7%)	33 (9%)
Total number of serious adverse events		25	34
	Infection	0	1 (<1%)
	Urinary retention	4 (1%)	7 (2%)
	Pain and bleeding	1 (<1%)	1 (<1%)
	Pain and stenosis	1 (<1%)	0
	Pain*	6 (2%)	10 (3%)
	Stenosis	1 (<1%)	0
	Constipation and urinary retention	0	2 (1%)
	Bleeding	6 (2%)	1 (<1%)
	Pain caused by fissure	1 (<1%)	2 (1%)
	Pain and constipation	0	1 (<1%)
	Constipation	0	3 (1%)
	Anaesthesia	0	2 (1%)
	Constipation and bleeding	0	2 (1%)
	Pain, constipation, and bleeding	0	1 (<1%)
	Difficulty passing urine	2 (1%)	0
	Haemorrhoids symptoms	1 (<1%)	0
	Fissure	1 (<1%)	0

Data are n (%).

* One participant in each group experienced two serious adverse events of pain.
