# Department of Error

**DOI:** 10.1016/S0140-6736(16)32059-1

**Published:** 2016-11-12

*Watson AJM, Hudson J, Wood J, et al, on behalf of the eTHoS study group. Comparison of stapled haemorrhoidopexy with traditional excisional surgery for haemorrhoidal disease (eTHoS): a pragmatic, multicentre, randomised controlled trial;* Lancet *2016; **388:** 2375–85*—In the Procedures section of this Article, the Haemorrhoid symptom score (HSS) range should be 0–26; the HSS score data in tables 1 and 2 and the reference to HSS data in the main text at the beginning of the fourth paragraph of the Results section on page 6 were incorrect; B Buckley should be added to the eTHoS study group, and the affiliations of M Jha and H Narula and A Fawole should be South Tees Hospitals NHS Foundation Trust, Middlesbrough, and The Mid Yorkshire Hospitals NHS Trust, Wakefield, respectively. These corrections have been made to the online version as of Oct 26, 2016, and the printed Article is correct.

