# Supplementary material for: Comparison of stapled haemorrhoidopexy with traditional excisional surgery for haemorrhoidal disease (eTHoS): a pragmatic, multicentre, randomised controlled trial
Source: Lancet. 2016 Nov 12;388(10058):2375–85. doi: 10.1016/S0140-6736(16)31803-7 (PMC5269572; doi:10.1016/S0140-6736(16)31803-7)
Supplement: Supplementary appendix [file mmc1.pdf]

THE LANCET

Supplementary appendix

This appendix formed part of the original submission and has been peer reviewed. We post it as supplied by the authors.

Supplement to: Watson AJM, Hudson J, Wood J, et al, on behalf of the eTHoS study group. Comparison of stapled haemorrhoidopexy with traditional excisional surgery for haemorrhoidal disease (eTHoS): a pragmatic, multicentre, randomised controlled trial. *Lancet* 2016; published online Oct 7. http://dx.doi.org/10.1016/S0140-6736(16)31803-7.

Table 1 Further secondary analyses of the primary outcome

	Stapled haemorrhoidopexy (n=388)	Traditional excisional surgery (n=386)	Mean difference	95% CI	p value
**Per-protocol analysis for participants with at least one short and one long term follow-up**					
6 weeks	0.225(0.151); 282	0.197(0.142); 299	0.029	(0.010 to 0.048)	0.0038
12 months	0.823(0.217); 240	0.827(0.219); 243	-0.009	(-0.040 to 0.022)	0.56
24 months	1.555(0.478); 251	1.623(0.418); 256	-0.078	(-0.153 to 0.002)	0.0447
**Multiple Imputation**					
**Participants that have either had surgery or at least one follow-up**					
6 weeks	354	358	0.024	(0.004 to 0.043)	0.0190
12 months	360	367	-0.012	(-0.038 to 0.014)	0.35
24 months	363	369	-0.063	(-0.111 to -0.015)	0.0115
**All participants randomised**					
6 weeks	388	386	0.020	(-0.002 to 0.042)	0.0704
12 months	388	386	-0.007	(-0.345 to 0.018)	0.56
24 months	388	386	-0.054	(-0.107 to -0.000)	0.0450

Data are mean (SD);N

Table 2. Subgroup analysis

	Stapled haemorrhoidopexy (n=388)	Traditional excisional surgery (n=386)	Mean difference	99% CI	p value
**Gender**					
Female	1.531(0.502); 147	1.626(0.414); 126			
Male	1.582(0.464); 148	1.612(0.446); 149	0.055	(-0.087 to 0.197)	0.30
**Grade of haemorrhoid**					
II	1.545(0.492); 56	1.532(0.467); 60			
III	1.564(0.483); 196	1.657(0.408); 171	-0.072	(-0.300 to 0.155)	0.40
IV	1.536(0.482); 43	1.585(0.458); 44	0.050	(-0.302 to 0.403)	0.**70**

Data are mean (SD);N

Table 3. Pain, analgesia and return to usual activities (further secondary outcomes)

	Stapled haemorrhoidopexy (n=388)	Traditional excisional surgery (n=386)	Effect size*	95% CI	p value
Pain in first week following surgery					
Number of patients followed-up	301(78%)	296(77%)			
Worst pain [Visual Analogue Scale]	6.9(2.5); 298	7.9(2.2); 295	- 1.04	(-1.59 to -0.49)	0.0006
Pain [Visual Analogue Scale]	4.0(2.5); 299	5.3(2.6); 296	- 1.35	(-1.81 to -0.89)	<0.0001
Analgesia	285(95%)	286(97%)	0.57	(0.27 to 1.19)	0.13
Number of days with analgesia	5.8(1.7); 285	6.4(1.3); 286	- 0.59	(-0.98 to -0.20)	0.0044
Pain at three weeks post-surgery					
Number of patients followed-up	291(75%)	282(73%)			
Pain [Visual Analogue Scale]	1.8(2.2); 290	2.6(2.5); 281	-0.72	(-1.16 to -0.28)	0.0026
Analgesia	111(38%)	145(51%)	0.58	(0.45 to 0.75)	<0.0001
Number of days with analgesia	4.3(2.1); 111	4.5(2.2); 145	-0.24	(-0.75 to 0.27)	0.34
Pain at six weeks post-surgery					
Number of patients followed-up	309(80%)	305(79%)			
Pain [Visual Analogue Scale]	1.3(2.0); 304	1.3(1.9); 301	-0.01	(-0.35 to 0.33)	0.96
Analgesia	73(24%)	67(22%)	1.11	(0.73 to 1.68)	0.62
Number of days in a week with analgesia	4.7(2.3); 72	4.0(2.3); 67	0.58	(-0.22 to 1.37)	0.15
Returned to usual activities after six weeks	276(89%)	271(89%)	1.05	(0.64 to 1.71)	0.85

Data are n (%) or mean (SD);N. *Effect size is mean difference except for Analgesia, this was the odds ratio.

Table 4. Total costs and QALYs gained at 24 months

	Costs	Mean difference	95% CI	QALY	Mean difference	95% CI	ICER
Stapled haemorrhoidopexy	941 (415)			1.62 (0.43)			Dominated
Traditional excisional surgery	602 (507)	337	(251 to 423)	1.69 (0.38)	-0.070	(-0.127 to -0.011)	

Data are mean(SD). QALYs =Quality adjusted life years. Dominated = Costs more and has less QALYs

Table 5. Participants included in the analysis at each time point

	Stapled haemorrhoidopexy (n=388)	Traditional excisional surgery (n=386)
Baseline	0.781(0.244);295	0.777(0.258);275
1 week	0.605(0.307);271	0.463(0.328);244
3 weeks	0.804(0.244);263	0.757(0.233);240
6 weeks	0.843(0.221);281	0.846(0.237);264
12 months	0.824(0.249);282	0.884(0.205);262
24 months	0.837(0.247);263	0.883(0.185);250

Data are mean(SD);N
